# Acquisition of a Novel Sulfur-Oxidizing Symbiont in the Gutless Marine Worm Inanidrilus exumae

**DOI:** 10.1128/AEM.02267-17

**Published:** 2018-03-19

**Authors:** C. Bergin, C. Wentrup, N. Brewig, A. Blazejak, C. Erséus, O. Giere, M. Schmid, P. De Wit, N. Dubilier

**Affiliations:** a Max Planck Institute for Marine Microbiology, Bremen, Germany; b Department of Biological and Environmental Sciences, University of Gothenburg, Göteborg, Sweden; c Biozentrum Grindel, Zoologisches Institut und Zoologisches Museum, Universität Hamburg, Hamburg, Germany; d Department of Microbiology and Ecosystem Science, Division of Microbial Ecology, University of Vienna, Vienna, Austria; e Department of Marine Sciences, University of Gothenburg, Tjärmö Marine Laboratory, Strömstad, Sweden; University of Bayreuth

**Keywords:** symbiosis, oligochaetes, Clitellata, chemoautotrophy, fluorescence *in situ* hybridization, 16S rRNA, *aprA*, *cbbL*, sulfur oxidizers, sulfate reducers, symbiont replacement/displacement

## Abstract

Gutless phallodrilines are marine annelid worms without a mouth or gut, which live in an obligate association with multiple bacterial endosymbionts that supply them with nutrition. In this study, we discovered an unusual symbiont community in the gutless phallodriline Inanidrilus exumae that differs markedly from the microbiomes of all 22 of the other host species examined. Comparative 16S rRNA gene sequence analysis and fluorescence *in situ* hybridization revealed that I. exumae harbors cooccurring gamma-, alpha-, and deltaproteobacterial symbionts, while all other known host species harbor gamma- and either alpha- or deltaproteobacterial symbionts. Surprisingly, the primary chemoautotrophic sulfur oxidizer “Candidatus Thiosymbion” that occurs in all other gutless phallodriline hosts does not appear to be present in I. exumae. Instead, I. exumae harbors a bacterial endosymbiont that resembles “*Ca*. Thiosymbion” morphologically and metabolically but originates from a novel lineage within the class Gammaproteobacteria. This endosymbiont, named Gamma 4 symbiont here, had a 16S rRNA gene sequence that differed by at least 7% from those of other free-living and symbiotic bacteria and by 10% from that of “*Ca*. Thiosymbion.” Sulfur globules in the Gamma 4 symbiont cells, as well as the presence of genes characteristic for autotrophy (*cbbL*) and sulfur oxidation (*aprA*), indicate that this symbiont is a chemoautotrophic sulfur oxidizer. Our results suggest that a novel lineage of free-living bacteria was able to establish a stable and specific association with I. exumae and appears to have displaced the “*Ca*. Thiosymbion” symbionts originally associated with these hosts.

**IMPORTANCE** All 22 gutless marine phallodriline species examined to date live in a highly specific association with endosymbiotic, chemoautotrophic sulfur oxidizers called “*Ca*. Thiosymbion.” These symbionts evolved from a single common ancestor and represent the ancestral trait for this host group. They are transmitted vertically and assumed to be in transition to becoming obligate endosymbionts. It is therefore surprising that despite this ancient, evolutionary relationship between phallodriline hosts and “*Ca*. Thiosymbion,” these symbionts are apparently no longer present in Inanidrilus exumae. They appear to have been displaced by a novel lineage of sulfur-oxidizing bacteria only very distantly related to “*Ca*. Thiosymbion.” Thus, this study highlights the remarkable plasticity of both animals and bacteria in establishing beneficial associations: the phallodriline hosts were able to acquire and maintain symbionts from two very different lineages of bacteria, while sulfur-oxidizing bacteria from two very distantly related lineages were able to independently establish symbiotic relationships with phallodriline hosts.

## INTRODUCTION

Symbioses are essential for the ecology and evolution of eukaryotes, but the processes involved in symbiosis initiation and maintenance are still only poorly understood (1, 2). Stable, long-lasting, and specific associations between symbionts and their hosts are common in vertically transmitted symbionts (inheritance of the symbiont from the parent). In such associations, if the symbionts are consistently and strictly transmitted to the host, codiversification occurs and is reflected in congruent phylogenies of the symbionts and their hosts (3, 4). However, strict vertical transmission over long evolutionary time periods, while well known from some insect symbioses, has rarely been observed in marine symbioses (4).

In the beneficial association between gutless marine phallodrilines (oligochaetes, Annelida, Clitellata, Naididae *sensu* Erséus et al.) (5) and their bacterial endosymbionts, the hosts lack a mouth, gut, and excretory system and are dependent on their symbionts for nutrition and waste recycling. The primary symbionts in all gutless phallodriline worms examined to date are large (2- to 7-μm) sulfur-storing members of the class Gammaproteobacteria, previously called Gamma 1 symbionts and now named “Candidatus Thiosymbion” (6). All individuals of a given host species share a highly similar “*Ca*. Thiosymbion” phylotype, with >99% 16S rRNA gene sequence similarity. Among host species, the 16S rRNA gene sequences of “*Ca*. Thiosymbion” are closely related to each other (>94.7% identity) and have evolved from a single common ancestor (6). Evidence for the chemoautotrophic metabolism of “*Ca*. Thiosymbion” includes the presence of sulfur globules (7), uptake experiments showing the incorporation of inorganic carbon (8, 9), immunohistochemical labeling of one of the key enzymes for CO_2_ fixation, i.e., ribulose-1,5-bisphosphate carboxylase/oxygenase (7, 10, 11), and more recently, metagenomic and proteomic analyses revealing the expression of pathways used for the fixation of inorganic carbon and the use of reduced sulfur compounds as an energy source (12, 13).

The primary “*Ca*. Thiosymbion” symbionts cooccur with secondary symbionts that are much smaller (0.7 to 1.5 μm), rod and coccus shaped, and belong to the Gamma-, *Delta*-, or Alphaproteobacteria, while other secondary symbionts, with an elongated, spiral-shaped morphotype, belong to the spirochetes (14). The secondary gammaproteobacterial symbionts are sulfur oxidizers, while the deltaproteobacterial symbionts are sulfate reducers. The sulfate-reducing symbionts provide the sulfur-oxidizing symbionts with reduced sulfur compounds, thus allowing their hosts to live in sediments with little or no environmental sulfide (11, 12, 15). The metabolism of the alphaproteobacterial and spirochete symbionts remains unclear (15, 16).

The dominant mode of symbiont transmission in gutless phallodrilines is vertical. Morphological studies indicated that both the primary and secondary symbionts are passed vertically from the parent worm to the offspring in a smear infection during the deposition of the egg in the sediment environment (17, 18). However, a recent analysis of “*Ca*. Thiosymbion” strains from 22 phallodriline host species found only weak congruence between symbiont and host phylogenies and little evidence for cospeciation (6). This indicates that repeated events of symbiont displacement through switching of “*Ca*. Thiosymbion” strains between host species have occurred in gutless phallodrilines (6).

In this study, we describe a gutless phallodriline in which “*Ca*. Thiosymbion” does not appear to be present, namely, Inanidrilus exumae Erséus, 2003, from the Bahamas (19, 20). We hypothesize that “*Ca*. Thiosymbion” was displaced in I. exumae, but not through host switching. Instead, these hosts appear to have taken up sulfur-oxidizing bacteria from a novel lineage only very distantly related to the ancestral “*Ca*. Thiosymbion” of gutless phallodrilines.

## RESULTS AND DISCUSSION

**FIG 1:**
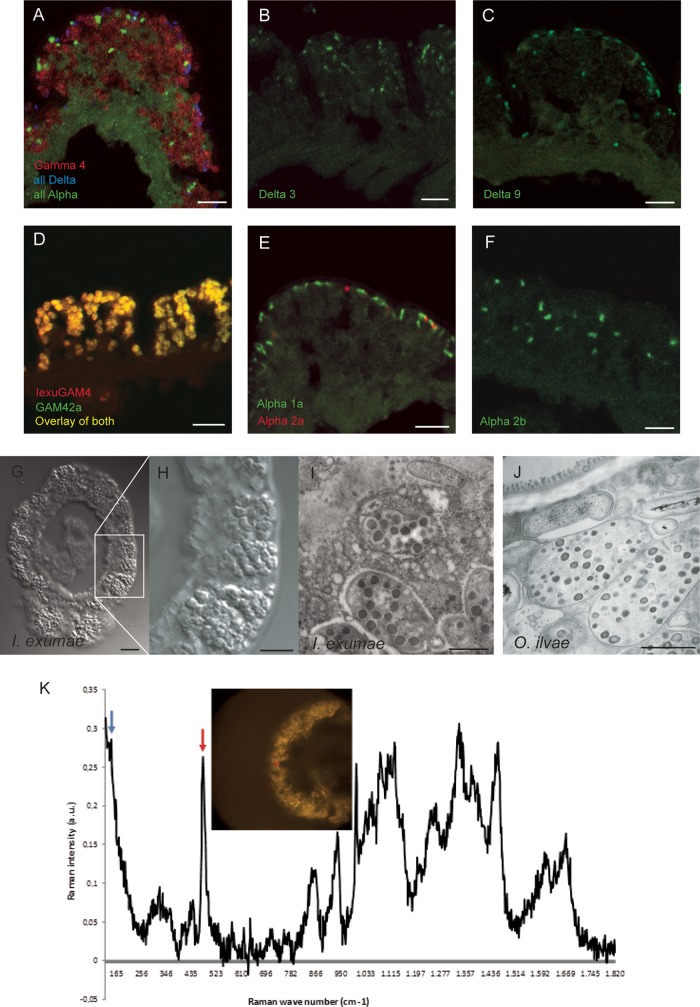
Bacterial symbionts in Inanidrilus exumae. (A to F) FISH images of the body wall of I. exumae. (A) The Gamma 4 symbionts (red, probe IexuGam4), deltaproteobacterial symbionts (blue, probe DSS658), and alphaproteobacterial symbionts (light green, combined probes ImakALF1b, IexuALFb, and IecuALFd) cooccur in the body wall of the worm. The symbiont-free parts of the worm's body wall are visible in green due to their high autofluorescence. (B) Delta 3 symbiont (green, probe Oalg/OilvDEL3). (C) Delta 9 symbiont (green, probe OalgDEL4). (D) Double hybridization with the Gamma 4 probe IexuGAM4 (red) and the general gammaproteobacterial probe GAM42a (green) shows a complete overlay of both probes (yellow), indicating that the Gamma 4 symbionts were the only Gammaproteobacteria present in I. exumae. (E) The Alpha 1a (green, probe IexuALFd) and Alpha 2a (red, probe ImakALF1b) symbionts always cooccurred in the two individuals examined. (F) The Alpha 2b symbiont (green, IexuALFb) was never observed to cooccur with the other alphaproteobacterial symbionts. (A to F) Scale bars, 5 μm. (G and H) Differential interference contrast images of I. exumae. (G) Cross section through an entire worm. The white box shows the part of the body wall shown at higher magnification in panel H. (H) The large Gamma 4 symbionts are visible in the body wall and fill the entire symbiont-containing region. (I) TEM image of I. exumae. The Gamma 4 symbionts have large, electron-dense globules, some of which contain sulfur, based on Raman analyses (see panel K and its legend, and the supplemental material as well), and have a morphology highly similar to that of “*Ca*. Thiosymbion” (see panel J). (J) TEM image of “*Ca*. Thiosymbion” in Olavius ilvae. (G to J) Scale bars, 10 μm (G), 5 μm (H), and 1 μm (I, J). (K) Results of Raman microspectroscopy. One clear sulfur peak is visible at 475 cm^−1^ in the symbiont-containing region of I. exumae. Raman spectra of host tissues without symbionts did not have a peak at 475 cm^−1^ or the two other peaks characteristic for S8 (and S6) sulfur (see Fig. S2) (57–60).

**FIG 2:**
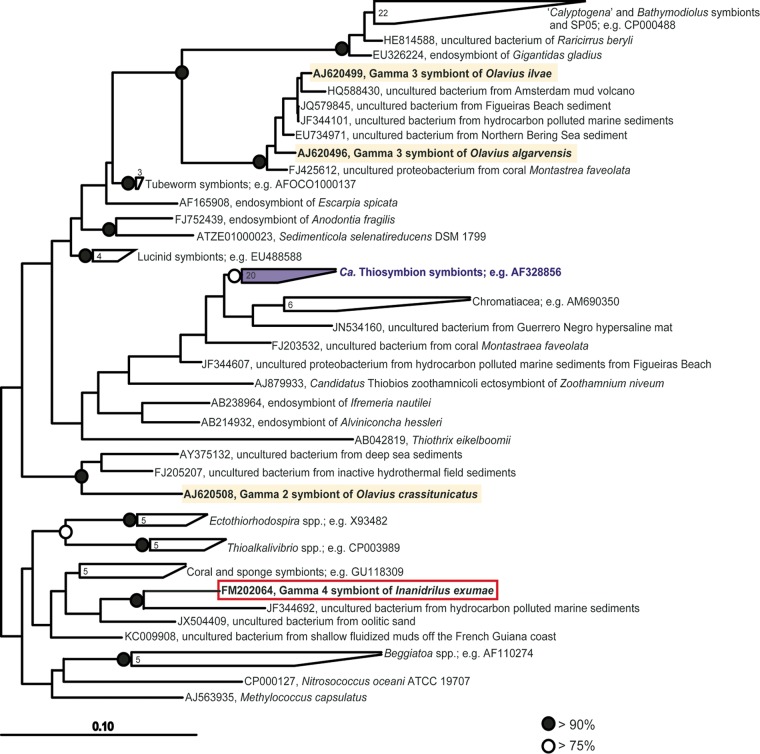
Phylogenetic analysis of the Gamma 4 symbiont of Inanidrilus exumae based on 16S rRNA gene sequences; GenBank accession numbers are shown. Sequences obtained in this study are framed with red boxes, sequences from gutless phallodriline symbionts are highlighted in yellow, and sequences belonging to the “*Ca*. Thiosymbion” clade are highlighted in purple. The consensus tree shown is based on maximum-likelihood analysis. Branching orders that were not supported by both calculation methods are shown as multifurcations; numbers within the polygons show the number of bacterial species concatenated in the node. Scale bars represent 10% estimated phylogenetic divergence for nonmultifurcated branches. Black- or white-filled circles indicate maximum-likelihood bootstrap values as indicated in the key.

Our morphological and molecular analyses revealed an unusual symbiotic community in Inanidrilus exumae, consisting of cooccurring gamma-, alpha-, and deltaproteobacterial symbionts (Fig. 1 and 2; see Fig. S1 in the supplemental material). In the five host species whose symbiont communities have been examined so far, alpha- and deltaproteobacterial symbionts appeared to be mutually exclusive (15, 16, 21, 22). Furthermore, we found no evidence for the presence of the primary symbiont, “*Ca*. Thiosymbion,” in I. exumae. This is surprising because the 22 gutless phallodriline species examined to date have always harbored “*Ca*. Thiosymbion” symbionts (6). In contrast, I. exumae harbors a sulfur oxidizer that resembles “*Ca*. Thiosymbion” in appearance and function but belongs to a lineage of Gammaproteobacteria not previously known to be associated with gutless phallodrilines or other eukaryotic hosts. In the following discussion, we will focus on the morphology, phylogeny, and potential function of this novel gammaproteobacterial symbiont of I. exumae. A brief description of the phylogenies and possible functions of the secondary alpha- and deltaproteobacterial symbionts of I. exumae is provided in the supplemental material.

### Morphology and phylogeny of the Gamma 4 symbiont.

Only a single gammaproteobacterial 16S rRNA phylotype, which we named Gamma 4, was found in the clone libraries from I. exumae worms (Table 1). Out of a total of 734 sequenced clones from seven host individuals, we never found a sequence that belonged to the “*Ca*. Thiosymbion” clade. In previous studies of other gutless oligochaete species, “*Ca*. Thiosymbion” could always be amplified with the general 16S rRNA primers we used in this study (8F and 1492R) (21, 22).

**TABLE 1:** Numbers of partial 16S rRNA, *aprA*, and *cbbL* gene sequences from cloned bacterial PCR products from Inanidrilus exumae

Gene	Source	Clone family/phylotype^*a*^	No. of sequences from I. exumae specimen no.:						
			1	2	3	4	5	6	7
16S rRNA gene	Gammaproteobacterial symbiont	Gamma 4	72	156	46	78	16	0	6
	Alphaproteobacterial symbionts	Alpha 1a	0	5	0	0	0	13	54
		Alpha 2a	0	0	5	0	0	13	11
		Alpha 2b	61	27	0	1	0	0	0
	Deltaproteobacterial symbionts	Delta 3	0	0	46	75	0	23	5
		Delta 9	5	1	0	0	0	0	0
	Associated bacteria	Delta 8	0	0	11	1	0	0	0
		Delta 10	0	0	0	3	0	0	0
*aprA*	Sulfur-oxidizing bacteria	AprA Ia	4	4	—^*b*^	—	—	—	—
		AprA Ib	2	5	—	—	—	—	—
		AprA IIa	7	11	—	—	—	—	—
		AprA IIb	11	4	—	—	—	—	—
	Sulfate-reducing bacteria		4	0	—	—	—	—	—
*cbbL*			—	—	—	—	—	29	18

a Sequences that shared >99% identity were grouped as a single phylotype. One or more clones of each phylotype and individual were sequenced in both directions for the almost-full-length 16S rRNA gene sequence and for partial *aprA* and *cbbL* gene sequences. SRB, sulfate-reducing bacteria.

b —, not analyzed.

Fluorescence *in situ* hybridization (FISH) provided further support for our assumption that the Gamma 4 symbiont is the only gammaproteobacterium present in I. exumae. FISH with a probe specific to the 16S rRNA gene sequence of the Gamma 4 symbiont (Table 2, IexuGAM4) showed that this sequence originated from large, oval-shaped bacteria (2 to 3 μm long and 1 to 2 μm wide) that were highly abundant and dominated the symbiont-containing region in all host individuals examined (Fig. 1A and D). Dual FISH hybridization with the specific IexuGAM4 probe and the general probe for Gammaproteobacteria (Table 2, GAM42a) showed a complete overlay of the hybridization signals, with both probes hybridizing in cells of the same large, oval-shaped morphotype (Fig. 1D). These results indicate that the Gamma 4 symbionts are the only Gammaproteobacteria present in I. exumae and that these hosts lack the “*Ca*. Thiosymbion” symbionts found in all other gutless phallodriline species examined.

**TABLE 2:** Symbiont-specific and general oligonucleotide probes used in this study

Probe	Target(s); specificity (sequence to which probe binds)	Probe sequence (5′–3′)	Position^*a*^	% of FA used^*b*^	Reference
NON338	Antisense, background control	ACT CCT ACG GGA GGC AGC	338–355	10–30	61
GAM42a	Gammaproteobacteria	GCC TTC CCA CAT CGT TT	1027–1043^*c*^	30–35	62
DSS658	I. exumae Delta 3 and Delta 9 symbionts, O. algarvensis and Olavius ilvae Delta 1 and Delta 3 symbionts, O. algarvensis Delta 4 symbiont, Desulfosarcina spp., Desulfofaba sp., Desulfococcus spp., Desulfofrigus spp.	TCC ACT TCC CTC TCC CAT	658–685	50–60	63
IexuGAM4	I. exumae Gamma 4 symbiont	ATT CCG CCT CCC TCT ACC GTA	657–1677	50	This study
IexuALFd	I. exumae Alpha 1a symbiont, Olavius loisae Alpha 1a-1 and Alpha 1a-2 symbionts, Inanidrilus leukodermatus Alpha 1a symbiont	GTA CCC GGC CAA ACC CGA	1131–1147	30	This study
ImakALF1b	I. exumae Alpha 2a symbiont, I. makropetalos Alpha 2 symbiont	TCC GGT CTC CGC GAC CCC	999–1014	35	22
IexuALFb	I. exumae Alpha 2b symbiont; DQ062742, EU133383, AJ810382, AY326603, DQ648967	TCT GGT CTC CGC GAC CGG	999–1014	30	This study
Oalg/OilvDEL3	I. exumae Delta 3 symbiont, O. algarvensis and O. ilvae Delta 3 symbionts	GTG CCT GCC TCC TGA AAG	1449–1465	30	15
OalgDEL4	I. exumae Delta 9 symbiont, O. algarvensis Delta 4 symbiont; AB121109, EF061975, DQ395063, EU290686, EU290687, DQ395004, DQ394892	GCC CAA CAA CTT CCG GTA	1427–1444	30	15

a Position in the 16S rRNA of Escherichia coli, unless otherwise noted.

b Percentage of formamide (FA) (vol/vol) used in the CARD-FISH hybridization buffer.

c Position in the 23S rRNA of E. coli.

Transmission electron microscopy (TEM) showed that the ultrastructure of the Gamma 4 symbionts was remarkably similar to that of “*Ca*. Thiosymbion” symbionts (Fig. 1I and J). Like “*Ca*. Thiosymbion,” the Gamma 4 symbiont was the largest (2- to 3-μm) and most abundant morphotype of the symbiotic community, and its cells were also filled with large, electron-dense globules (Fig. 1I).

Comparative phylogenetic analyses of 16S rRNA gene sequences revealed that the Gamma 4 symbiont belongs to a novel lineage of Gammaproteobacteria not previously shown to be associated with animal or plant hosts (Fig. 2). While the phylogenetic resolution of the gammaproteobacterial tree was not well defined at the basal nodes (Fig. 2), we never observed any clustering of the Gamma 4 symbiont sequence with the “*Ca*. Thiosymbion” clade in our analyses. Indeed, the 16S rRNA gene sequence of the Gamma 4 symbiont differed from sequences belonging to the “*Ca*. Thiosymbion” clade by more than 10% (Fig. 2). The closest uncultured relative, with a sequence divergence of 7%, was a sediment clone from a beach in the Cíes Islands off the coast of northern Spain (GenBank accession number JF344692). The closest cultured relatives, with sequence divergences ranging from 9 to 10%, were sulfur-storing members of the family Ectothiorhodospiraceae and bacteria from the genera Nitrosococcus and Methylococcus.

### Indications for autotrophic sulfur oxidation by the Gamma 4 symbiont.

**FIG 3:**
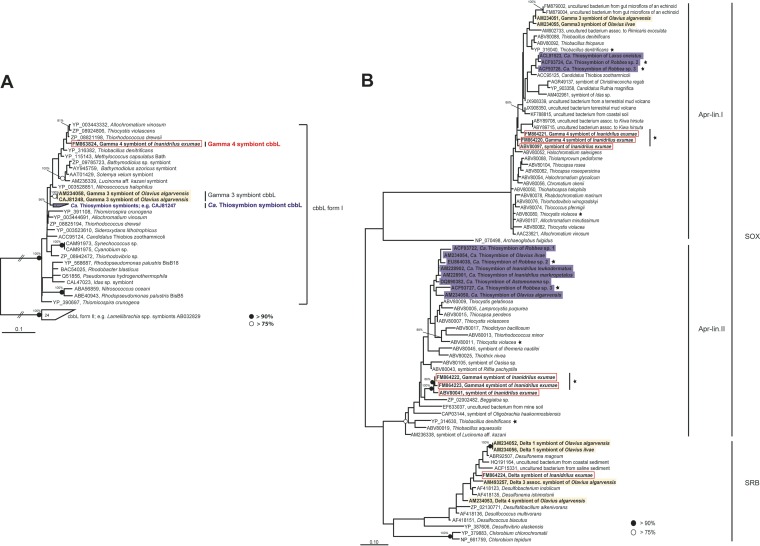
Phylogenetic affiliations of *cbbL*, encoding the large subunit of RubisCO form I (A), and APS reductase *aprA* (B) sequences from Inanidrilus exumae, based on deduced amino acid sequences. Based on close phylogenetic relationships to free-living and symbiotic sulfur-oxidizing bacteria, we assume that the *cbbL* sequence and the *aprA* sequences from AprA lineages I and II originated from the Gamma 4 symbiont, while a fifth *aprA* sequence most likely originated from a deltaproteobacterial symbiont. Asterisks show bacteria that have *aprA* gene sequences from both lineage I and II. Sequences obtained in this study are framed with a red box, sequences from “*Ca*. Thiosymbion” are highlighted in purple, and sequences from other gutless phallodriline symbionts are highlighted in yellow. GenBank accession numbers are shown. Scale bars represent 10% estimated phylogenetic divergence for nonmultifurcated branches. Numbers in the polygons show the number of bacterial species concatenated in the node. Black- or white-filled circles indicate maximum-likelihood bootstrap values as indicated in the keys, while percentages show posterior probabilities from Bayesian inference.

Despite their divergent phylogenies, “*Ca*. Thiosymbion” and the I. exumae Gamma 4 symbionts not only share highly similar morphologies but also appear to have similar functional roles as chemoautotrophic sulfur oxidizers. As shown for other chemoautotrophic symbioses, the *cbbL* gene, coding for one of the key proteins of the Calvin-Benson-Bassham (CBB) cycle, the ribulose-1,5-bisphosphate carboxylase/oxygenase (RubisCO) form I large subunit, was present in I. exumae (Fig. 3A and Table 1) (23). The *cbbL* sequence obtained from I. exumae grouped with sequences from other gammaproteobacterial chemoautotrophs, such as free-living Chromatiaceae and sulfur-oxidizing symbionts from other marine invertebrates. It is therefore likely that the I. exumae *cbbL* sequence originated from the I. exumae Gamma 4 symbiont.

Evidence for the potential of the Gamma 4 symbiont to oxidize reduced sulfur compounds was provided by Raman spectroscopy analyses, which revealed sulfur in the cells of these symbionts (Fig. 1K; Fig. S2.1 and S2.2). Moreover, we amplified *aprA* genes (encoding AprA, the alpha subunit of adenosine-5′-phosphosulfate [APS] reductase) related to those of free-living and symbiotic sulfur-oxidizing bacteria from I. exumae individuals (Fig. 3B and Table 1). Sequences belonging to two phylogenetically distinct APS reductase lineages, AprA I and II, were found in I. exumae (Fig. 3B). We assume that the sequences from both AprA I and II originated from the Gamma 4 symbiont, as no other gammaproteobacterial sulfur oxidizers were found in I. exumae and the alphaproteobacterial symbionts of gutless phallodrilines do not appear to have an APS reductase (22). The presence of two gene loci for AprA has been shown for several free-living sulfur-oxidizing bacteria and is therefore not unusual (24). Meyer and Kuever (24) hypothesized that the presence of two gene loci might provide physiological versatility in habitats with oscillating oxygen and sulfide concentrations. This may well be the case for I. exumae and other gutless phallodrilines, which migrate between upper, oxidized and lower, sulfidic sediment layers.

### Symbiont replacement in I. exumae?

**FIG 4:**
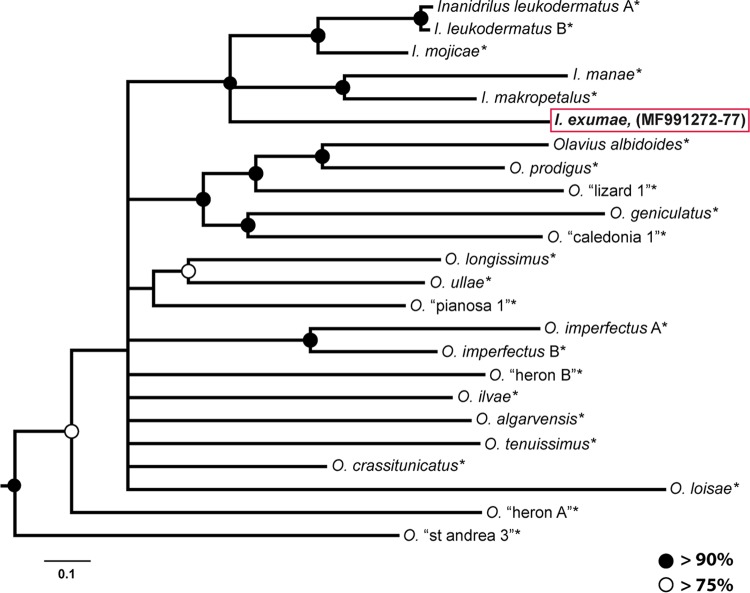
Phylogenetic tree of gutless phallodrilines using Bayesian inference analysis of six concatenated genetic markers for the host (mt12S, mt16S, 18S, 28S rRNA, mtCOI, and ITS genes). Posterior probability values are indicated at nodes. Scale bar represents 10% estimated phylogenetic divergence for nonmultifurcated branches. GenBank accession numbers for I. exumae sequences are given in the figure and under “Accession number(s)” in the text. GenBank accession numbers for species with asterisks are as follows: 18S rRNA, KP943792 to KP943817; 28S rRNA, KP943818 to KP943844; mtCOI, KP943845 to KP943866; ITS, KP943867 to KP943884; mt12S rRNA, KP943885 to KP943908; and mt16S rRNA, KP943909 to KP943931.

What are the evolutionary events that might explain the presence of a novel sulfur-oxidizing symbiont and the absence of the ubiquitous “*Ca*. Thiosymbion” in I. exumae? “*Ca*. Thiosymbion” is present in all 22 gutless phallodriline species examined to date from habitats around the world, including six host species from the Bahamas, some of which cooccur with I. exumae (6, 20). All “*Ca*. Thiosymbion” 16S rRNA gene sequences are closely related to each other and belong to a monophyletic clade (6). The phallodriline hosts have also evolved from a single common ancestor, based on morphological (25, 26) and molecular data (27, 28). Furthermore, I. exumae is not an early-diverging or basal species within the gutless phallodrilines but, rather, closely related to other Inanidrilus species, which form a monophyletic group within the gutless phallodrilines (Fig. 4). Since all gutless phallodrilines, including the four Inanidrilus species closely related to I. exumae (Fig. 4), harbor “*Ca*. Thiosymbion” symbionts (6, 14), the most parsimonious conclusion is that the ancestor of I. exumae also harbored a “*Ca*. Thiosymbion” symbiont.

How could the Gamma 4 symbiont have displaced “*Ca*. Thiosymbion” in I. exumae? We envision the following three successive scenarios that could explain how the ancestral symbiont of I. exumae was displaced. In the first step, when “*Ca*. Thiosymbion” was still the primary symbiont, the ancestors of the Gamma 4 symbiont must have been able to enter and persist in I. exumae at some point in their evolutionary history. The early developmental stages of the worms were the most likely window of opportunity for infection by bacteria from the environment. Gutless phallodrilines lay single eggs into the surrounding sediment. The egg remains attached to the parent worm and is fertilized with sperm and coated with symbiotic bacteria from the parent worm in a smear infection and then encased in a cocoon, which is eventually deposited in the sediment (17, 18). Free-living bacteria from the sediment could easily become encased within the cocoon during this process and colonize the developing embryo.

During a second, transition phase, the Gamma 4 bacteria and the “*Ca*. Thiosymbion” symbiont may have coexisted in I. exumae. In some gutless phallodriline species, “*Ca*. Thiosymbion” cooccurs with secondary sulfur-oxidizing Gammaproteobacteria, called Gamma 2 and 3 symbionts (15, 16). However, these secondary sulfur-oxidizing symbionts are much smaller than “*Ca*. Thiosymbion” and occur in the small interstitial spaces between the large “*Ca*. Thiosymbion” cells, so that competition for space does not appear to occur. Also, they have functional differences that may allow niche separation: the Gamma 3 symbiont of Olavius algarvensis, for example, uses nitrate as an electron acceptor, while “*Ca*. Thiosymbion” uses oxygen (12, 13). Furthermore, the O. algarvensis Gamma 3 symbiont can use additional electron donors, such as carbon monoxide, which cannot be used by its “*Ca*. Thiosymbion” symbiont, thereby reducing competition for energy sources (13, 29).

In the third and final step of displacement, the Gamma 4 symbiont appears to have outcompeted “*Ca*. Thiosymbion” in I. exumae, at least in the host population we examined (16 individuals from the same collection site were examined with molecular methods or FISH). While it is possible that our methods were not sensitive enough to detect residual, very low numbers of “*Ca*. Thiosymbion” cells in the I. exumae individuals we examined, these hosts were clearly dominated by Gamma 4 symbionts. The niche separation between “*Ca*. Thiosymbion” and Gamma 4 symbionts may not have been sufficient to allow their codominance in I. exumae. However, other factors could also explain the displacement of “*Ca*. Thiosymbion,” such as a massive viral infection event, a strong competitive advantage of the Gamma 4 symbiont over “*Ca*. Thiosymbion,” or harmful mutations in the ancestral “*Ca*. Thiosymbion” population.

Recent studies have shown that symbiont displacement is not as rare as previously assumed. Even in associations in which vertical transmission of symbionts occurs over long evolutionary times, acquisition of symbionts from novel lineages of environmental bacteria and symbiont displacement can occur occasionally in both aquatic and terrestrial symbioses (30–38). In the gutless phallodriline symbioses, Zimmermann et al. (6) revealed that displacement of “*Ca*. Thiosymbion” may have occurred numerous times. However, in the 22 phallodriline species analyzed by Zimmermann et al. (6), displacement appears to have always occurred within the “*Ca*. Thiosymbion” clade; that is, the ancestral “*Ca*. Thiosymbion” strain of a given host species was displaced by a “*Ca*. Thiosymbion” strain from another host species. I. exumae is the only species in which we found indications for the displacement of “*Ca*. Thiosymbion” by a novel, phylogenetically distinct lineage of bacteria not closely related to “*Ca*. Thiosymbion.” Genomic, transcriptomic, and proteomic analyses of the Gamma 4 symbionts are needed to better understand the factors that allowed these bacteria to successfully colonize and persist in I. exumae.

## MATERIALS AND METHODS

### Site description and specimen collection.

Inanidrilus exumae specimens were collected from shallow water sediments off Lee Stocking Island, Bahamas, in April 1999. I. exumae cooccurred with several other gutless phallodriline species in a water depth of about 3 m in sediments that were largely composed of fine calcareous sands (20). The worms were extracted by decantation and identified under a microscope. In total, 16 specimens were divided for different analyses: 8 were fixed in 80% ethanol for DNA extraction (7 for analysis of bacterial genes and 1 for analysis of host genes), and another 8 were cut and fixed either for TEM or for FISH as described previously (16, 39). Samples were stored at 4°C.

### DNA preparation and PCR amplification.

For DNA extraction and subsequent PCR of bacterial genes, seven individual worms were prepared singly. Specimens were rinsed in MilliQ water, and DNA was isolated as described previously (6, 21), following the method of Schizas and colleagues (40). The bacterial 16S rRNA genes were amplified with primers specific for the bacterial 16S rRNA gene 8F and 1492R (41) using *Taq* DNA polymerase (Eppendorf, Hamburg, Germany). The bacterial 16S rRNA genes from I. exumae individuals 1 and 2 were amplified by applying the reconditioning approach (42, 43) under the following conditions: initial denaturation at 96°C for 5 min, 15 plus 5 and 15 plus 7 cycles for I. exumae 1 and I. exumae 2, respectively, at 96°C for 1 min, 44°C for 2 min, and 72°C for 3 min, followed by a final elongation of 10 min at 72°C. The PCR conditions for I. exumae individuals 3, 4, and 5 were as described previously (22). The PCR conditions for I. exumae individuals 6 and 7 were initial denaturation at 94°C for 5 min, 30 cycles at 94°C for 1 min, 42°C for 1.5 min, and 72°C for 2 min, followed by a final elongation of 30 min at 72°C. The PCR protocols differed due to protocol improvements in the course of our biodiversity studies during the last decade and sample availability.

Genes coding for RubisCO form I and APS reductase were PCR amplified with 30 and 33 cycles, respectively. The following primers were used: cbbLF (5′-CACCTGGACCACVGTBTGG-3′) and cbbLR (5′-CGGTGYATGTGCAGCAGCATICCG-3′) for *cbbL* (22) and aps1F (5′-TGGCAGATCATGATYMAYGG-3′) and aps4R (5′-GCGCCAACYGGRCCRTA-3′) for *aprA*, with the annealing temperature at 60°C for *aprA* and 48°C for *cbbL* (22).

Host genes were amplified and sequenced from DNA extracted from a single I. exumae individual (sample CE73) as previously described (6).

### Cloning and sequencing.

PCR products for all bacterial genes (16S rRNA, *cbbL*, and *aprA*) were cloned separately for each individual worm using the pCR4-TOPO plasmids and TOP10 chemically competent cells (Invitrogen, Carlsbad, CA) according to the manufacturer's protocol. Clones were selected for the correct insert size and sequenced, and sequences grouped in clone groups as described in reference 44. PCR products for amplified host genes were sequenced directly.

### Phylogenetic analyses of symbiont sequences.

Sequences were checked with BLAST (45, 46) for similarity searches. Chimeras were identified using CHIMERA_CHECK from the Ribosomal Database Project (RDP) (47) and manually in sequence alignments and were excluded from further analysis.

Sequences were trimmed at the 5′ and 3′ ends, and only nearly full-length 16S rRNA gene sequences, including outgroup sequences, were considered for tree calculations (>1,200 bp) using the ARB software package (48) and SILVA SSU Ref, release_NR99_119 July 2014 (49). The sequence similarities of the nucleotide sequences were calculated by distance matrix analysis, excluding the primer region. Phylogenetic trees for 16S rRNA gene sequences were calculated using Bayesian inference (MrBayes version 3.2) (50) and maximum-likelihood (ML)-based methods (PHYML) provided within the ARB software package as described previously (6). We used the generalized time reversible (GTR) substitution model for both analyses. Trees for alpha-, gamma-, and deltaproteobacterial symbionts were calculated separately, and consensus trees were constructed based on the information from the Bayesian inference and maximum-likelihood analyses. Node stability was evaluated using posterior probabilities (Bayesian inference).

The phylogenies of the *aprA* and *cbbL* genes were generated from partial sequences of deduced amino acid sequences, with 134 and 101 amino acid positions compared, respectively. Sequences for each gene were aligned separately using MAFFT, provided within the ARB software package, and the 5′ and 3′ ends trimmed. For phylogenetic tree reconstruction, we used maximum-likelihood analyses (PHYML with LG and RAxML with JTT) and the bootstrapping algorithm in RaxML (51), as well as Bayesian inference (50). For the Bayesian inference analyses, the optimal model of amino acid evolution for AprA and CbbL was determined using ProtTest3 (https://github.com/ddarriba/prottest3) (LG+G for both proteins). The protein alignments were imported into MrBayes version 3.2 and run in duplicate runs with four chains each (one hot and three cold) until convergence (26 million generations for AprA and 50 million generations for CbbL). Trees were sampled every 1,000 generations and were then summarized in a majority rule consensus using a burn-in value of 20%. Clade posterior probabilities were plotted onto the ML trees shown in Fig. 3.

### Phallodriline host phylogeny.

The mitochondrial 12S (mt12S), mt16S, and mtCOI, nuclear 18S and 28S rRNA, and ITS genes of 22 gutless phallodrilines and 5 gut-bearing annelids submitted by Zimmermann et al. (6) and the genes from I. exumae [see “Accession number(s)” below] were used for phylogenetic reconstruction. Sequences for each gene were aligned separately using MAFFT version 7 (52) with the Q-INS-I setting (53), alignments were manually adjusted, and the 5′ and 3′ ends trimmed using BioEdit as described in Zimmermann et al. (6).

The optimal substitution model for each alignment was assessed, and phylogenetic trees were reconstructed using Bayesian inference (MrBayes version 3.2) (50) as described previously (6). Node stability was evaluated using posterior probabilities (Bayesian inference) and bootstrap support (100 RaxML rapid bootstrap runs), with values above 0.80 considered significant.

### FISH.

Parts of eight I. exumae individuals were fixed and prepared for fluorescence *in situ* hybridization (FISH) as described previously (16), with the slight modification that we used xylol instead of Roti-Histol (Carl Roth, Karlsruhe, Germany). Symbionts were detected by catalyzed reporter deposition (CARD)-FISH as described previously (54), with slight modifications as follows. Tissue sections were hybridized with the horseradish peroxidase (HRP)-labeled probe for 2.5 h at 46°C. After washing for 15 min at 48°C in washing buffer, the sections were equilibrated for 20 min at room temperature in phosphate-buffered saline (PBS; pH 8.0). The moist tissue sections were incubated with amplification solution (1× PBS, pH 8.0, 2 M NaCl, 0.1% blocking reagent in 100 mM maleic acid buffer, pH 7.5, 0.0015% [vol/vol] H_2_O_2_, and 1% Alexa Fluor 488, 546, or 633 dye [Molecular Probes, Leiden, The Netherlands]) for 30 min at 46°C in the dark and rinsed in 1× PBS buffer for at least 20 min at room temperature. For dual and triple hybridizations, the CARD-FISH protocol was repeated two or three times on the same sections using different probes and Alexa Fluor dyes, and the HRP was inactivated after each hybridization round by using 0.01 M HCI for 10 min at room temperature after the last washing step (16).

The oligonucleotide probes and formamide concentrations used in this study are listed in Table 2. Probes designed with ARB were checked for in silico specificity against sequences in GenBank using BLAST and against rRNA sequence databases using ProbeCheck (55). The specificity was also tested experimentally against mismatched 16S rRNA gene sequences of either reference strains or symbionts. General probes for Bacteria (EUB338 I to III), Gammaproteobacteria (GAM42a), and a subgroup of the Deltaproteobacteria (DSS658) were used as positive controls, and the antisense probe NON338 was used as a negative control. All hybridizations were performed at formamide concentrations ensuring the highest possible specificity.

### TEM.

Parts of eight I. exumae worms were fixed for transmission electron microscopy (TEM), washed in 0.05 M NA-cacodylate, and postfixed in osmium tetroxide. After dehydration in an acetone series, specimens were embedded in Spurr resin (56), and the worms' middle parts, containing the symbiont region, sectioned on an ultramicrotome. For electron microscopy, ultrathin sections were stained with uranyl acetate and lead citrate and examined with a Zeiss EM 902A (39).

### Raman spectroscopy.

Raman spectroscopy was done on parts of two of the individuals used for FISH analyses as described in Eichinger et al. (57). More details on material and methods, as well as results and discussion, can be found in the supplemental material.

### Accession number(s).

All sequences obtained in this study were submitted to GenBank and are available under the accession numbers given here. Inanidrilus exumae bacterial symbiont gene sequences include the following. Gamma 4 symbiont sequences: 16S rRNA gene, FM202064; *cbbL*, FM863824; and *aprA* lineages I and II, FM864220 to FM864223. Delta symbiont *aprA*, FM864224. 16S rRNA gene sequences for other symbionts: Delta 3 symbiont, FM202060; Delta 9 symbiont, FM202059; Delta 8-associated bacterium, FM202066; Delta 10-associated bacterium, FM202065; Alpha 1a symbiont, FM202063; Alpha 2b symbiont, FM202062; and Alpha 2a symbiont, FM202061. Host genes from I. exumae sample CE73 are as follows: mt12S gene, MF991272; mt16S gene, MF991273; 18S gene, MF991275; 28S gene, MF991276; mtCOI gene, MF991274; and ITS gene, MF991277. Other accession numbers are given in figures and in Table 2.

## Supplementary Material

Supplemental material
