# Supplementary material for: Acquisition of a Novel Sulfur-Oxidizing Symbiont in the Gutless Marine Worm Inanidrilus exumae
Source: Appl Environ Microbiol. 2018 Mar 19;84(7):e02267-17. doi: 10.1128/AEM.02267-17 (PMC5861843; doi:10.1128/AEM.02267-17)
Supplement: Supplemental material [file supp_84_7_e02267-17__index.html]

Supplemental material 

# Acquisition of a Novel Sulfur-Oxidizing Symbiont in the Gutless Marine Worm Inanidrilus exumae

## Supplemental material

- Supplemental file 1 -

  Supplemental materials and methods; phylogenetic analysis (Fig. S1); Raman spectrogram (Fig. S2).

  PDF, 1.4M
