# Supplementary material for: Acquisition of a Novel Sulfur-Oxidizing Symbiont in the Gutless Marine Worm Inanidrilus exumae
Source: Appl Environ Microbiol. 2018 Mar 19;84(7):e02267-17. doi: 10.1128/AEM.02267-17 (PMC5861843; doi:10.1128/AEM.02267-17)

**Bergin et al.**

## Supplemental Material

### Co-occurring alpha- and deltaproteobacterial symbionts in *I. exumae*

Clone libraries of the 16S rRNA gene from seven *I. exumae* individuals contained sequences belonging to the Gamma-, Alpha- and Deltaproteobacteria (Table 1). Of the four deltaproteobacterial phylotypes found in the clone libraries, the Delta 3 and Delta 9 could be identified as symbionts using FISH with probes specific to these sequences (Table 2). These symbionts were small, rod-shaped bacteria that occurred mostly in the periphery of the symbiont containing region just below the worm's cuticle (Fig. 1A - C). In phylogenetic analyses, both deltaproteobacterial symbionts belonged to the *Desulfobacteraceae* (Fig. S1A). Two further deltaproteobacterial 16S rRNA gene phylotypes (named Delta 8 and 10) were found in the *I. exumae* clone libraries (Table 1, Figure S1A), but probes specific to these sequences *in silico* did not show FISH signals. Explanations for the absence of signals from these probes include a) very low abundances of the Delta 8 and 9 bacteria in the two *I. exumae* individuals examined, b) the sequences originated from contaminants outside of the worm, or c) the probes, although predicted to target an easily accessible region of the 16S rRNA (1), did not hybridize properly.

The close phylogenetic relationship of the Delta 3 and 9 symbionts of *I. exumae* to sulfate-reducing symbionts of other gutless phallodrilines and free-living sulfate reducers suggest that the *I. exumae* deltaproteobacterial symbionts are also sulfate reducers. This conclusion is supported by the presence of an *aprA* gene in *I. exumae* that fell within the

lineage of AprA sequences from sulfate-reducing prokaryotes (Fig. 3B). Given the presence of at least two deltaproteobacterial symbionts in *I. exumae* with close relationships to sulfate-reducing bacteria, we would have expected to find more than one deltaproteobacterial *aprA* gene sequence. Unfortunately, we did not have enough material for additional analyses of functional genes.

The close phylogenetic relationship of the *I. exumae* deltaproteobacterial symbionts to those of other gutless phallodriline symbionts and free-living sulfate-reducing bacteria suggests that these fulfil a similar role as the deltaproteobacterial symbionts of the gutless phallodrilines *O. algarvensis* and *O. ilvae* from the Mediterranean Sea. In these Mediterranean worms, the sulfate-reducing deltaproteobacterial symbionts produce reduced sulfur compounds, which are used as an energy source by the gammaproteobacterial sulfur-oxidizing symbionts, thereby contributing to an internal sulfur cycle in a sulfide-poor habitat (2–5).

In addition to deltaproteobacterial symbionts, we found three alphaproteobacterial 16S rRNA phylotypes in *I. exumae* clone libraries, Alpha 1a, Alpha 2a, and Alpha 2b (Table 1). FISH with probes specific to these phylotypes confirmed that all three originated from symbionts in *I. exumae* (Table 2, Fig. 1E and F). All three alphaproteobacterial symbionts were most closely related to symbionts from other gutless phallodriline species (Fig. S1B). The closest cultured relatives were *Magnetovibrio blakemorei* and *Pelagibius litoralis*. All but one out of the seven examined *I. exumae* individuals harboured at least one alphaproteobacterial symbiont based on 16S rRNA gene sequencing, indicating that these alphaproteobacterial symbionts are important if not

essential for the association. However, as with the alphaproteobacterial symbionts of other gutless phallodrilines, their role in the association remains as yet unclear (6, 7).

We found some variation in the relative number of 16S rRNA gene sequences from the alpha- and deltaproteobacterial symbionts in the seven *I. exumae* individuals examined in this study (Table 1). However, given the low abundances of clones from these symbionts, and possible bias due to PCR amplification, the true composition of the symbiont community in these worms remains unclear. In depth sequencing using unbiased approaches is needed to resolve if the gamma-, alpha-, and deltaproteobacterial symbionts always co-occur in all host individuals, and to resolve their relative abundances within single host individuals as well as within the host population as a whole.

In the six other phallodriline host species whose secondary symbiont communities have been examined in depth (two *Inanidrilus* and four *Olavius* species), either alpha- or deltaproteobacterial symbionts co-occurred with *Ca.* Thiosymbion (4, 6–8). We assumed that sediment type influences the distribution of these secondary symbionts, because we found alphaproteobacterial symbionts only in hosts from biogenic calcareous sediments such as the Bahamas (6, 7) while deltaproteobacterial symbionts were found in hosts from non-biogenic silicate sediments (4, 9). This study shows that there are exceptions to this habitat pattern: alpha- and deltaproteobacterial symbionts do not mutually exclude each other; they might rather complement each other or interact beneficially with each other to the advantage of the symbiosis.

**Raman spectroscopy of *I. exumae***

We used Raman spectroscopy to investigate if *I. exumae* Gamma 4 symbionts have sulfur inclusions in their cells, to provide additional support for the sulfur-oxidizing metabolism of these bacteria. Raman spectroscopy was done as described in Eichinger et al. 2011 (10) on the same two *I. exumae* individuals used for FISH analyses.

For comparison of the Raman spectra from *I. exumae* Gamma 4 symbiont to those of *Ca.* Thiosymbion, we used the gutless phallodriline *Olavius* sp. from Elba. We homogenated *Olavius* sp. individuals and analysed PFA-fixed and unfixed, fresh *Ca.* Thiosymbion cells from this host species. Samples were placed under a confocal LabRAM HR800 Raman microspectrometer (Horiba, Germany) equipped with a 50-mW 532.17-nm laser. Cells for Raman analysis were chosen in the live-view mode of the Labspec software, ver. 5.25.15 (Horiba). Exposure times and acquired spectra are specified in the respective figure legend (Fig. S2). Raman spectra were baseline corrected, normalized, and exported to a file format readable by Excel (Microsoft).

Raman spectra of fresh *Ca*. Thiosymbion had all three peaks characteristic for S8 sulfur (154 $cm^{-1}$, 216 $cm^{-1}$ and 474 $cm^{-1}$ (11, 12)) (Fig. S2.4). In fixed *Ca*. Thiosymbion cells, only one sulfur peak at 480 $cm^{-1}$ could be detected (Fig. S2.5). Sulfur peaks decreased over time in fresh samples: After one day we found only one peak at 480 $cm^{-1}$, and after two days none of the three sulfur peaks could be detected (data not shown).

The only *I. exumae* material available for Raman analysis were two specimens that had been fixed for FISH and embedded in paraffin (see Material and Methods in main paper). The paraffin blocks with the worms were sectioned with a microtome and the sections placed on uncoated glass slides or $CaF_2$ slides. We dewaxed the worm sections

with xylene and ethanol as described previously (8) because high background peaks from the paraffin masked the sulfur peaks.

We identified a clear sulfur peak at about 475 $cm^{-1}$ in the symbiont-containing region of the two examined *I. exumae* individuals (Fig. S2.1 - 2.2). Raman spectra of host tissues without symbionts did not have a peak at 475 $cm^{-1}$ or the two other peaks characteristic for S8 or S6 sulfur (Fig. S2.3). These results indicate that bacteria in the symbiont-containing region of *I. exumae* contained sulfur. Given that only the Gamma 4 symbiont had sulfur vesicles based on our TEM analyses, it is likely that the sulfur found with Raman spectroscopy originated from the Gamma 4 symbionts.

**References**

1. Behrens S, Ruhland C, Inacio J, Huber H, Fonseca A, Spencer-Martins I, Fuchs BM, Amann R. 2003. In situ accessibility of small-subunit rRNA of members of the domains Bacteria, Archaea, and Eucarya to Cy3-labeled oligonucleotide probes. Appl Environ Microbiol 69:1748–1758.

2. Dubilier N, Mulders C, Ferdelman T, de Beer D, Pernthaler A, Klein M, Wagner M, Erséus C, Thiermann F, Krieger J, Giere O, Amann R. 2001. Endosymbiotic sulphate-reducing and sulphide-oxidizing bacteria in an oligochaete worm. Nature 411:298–302.

3. Woyke T, Teeling H, Ivanova NN, Huntemann M, Richter M, Gloeckner FO, Boffelli D, Anderson IJ, Barry KW, Shapiro HJ, Szeto E, Kyrpides NC, Mussmann M, Amann R, Bergin C, Ruehland C, Rubin EM, Dubilier N. 2006. Symbiosis insights through metagenomic analysis of a microbial consortium. Nature 443:950–955.

4. Ruehland C, Blazejak A, Lott C, Loy A, Erséus C, Dubilier N. 2008. Multiple bacterial symbionts in two species of co-occurring gutless oligochaete worms from Mediterranean sea grass sediments. Environ Microbiol 10:3404–3416.

5. Kleiner M, Wentrup C, Lott C, Teeling H, Wetzel S, Young J, Chang Y-J, Shah M, VerBerkmoes NC, Zarzycki J, Fuchs G, Markert S, Hempel K, Voigt B, Becher D, Liebeke M, Lalk M, Albrecht D, Hecker M, Schweder T, Dubilier N. 2012. Metaproteomics of a gutless marine worm and its symbiotic microbial community reveal unusual pathways for carbon and energy use. Proc Natl Acad Sci 109:E1173–E1182.

6. Dubilier N, Amann R, Erséus C, Muyzer G, Park SY, Giere O, Cavanaugh CM. 1999. Phylogenetic diversity of bacterial endosymbionts in the gutless marine oligochete *Olavius loisae* (Annelida). Mar Ecol Prog Ser 178:271–280.

7. Blazejak A, Kuever J, Erséus C, Amann R, Dubilier N. 2006. Phylogeny of 16S rRNA, ribulose 1,5-risphosphate carboxylase/oxygenase, and adenosine 5′-phosphosulfate reductase genes from gamma- and alphaproteobacterial symbionts in gutless marine worms (Oligochaeta) from Bermuda and the Bahamas. Appl Environ Microbiol 72:5527–5536.

8. Blazejak A, Erséus C, Amann R, Dubilier N. 2005. Coexistence of bacterial sulfide oxidizers, sulfate reducers, and spirochetes in a gutless worm (Oligochaeta) from the Peru margin. Appl Environ Microbiol 71:1553–1561.

9. Dubilier N, Blazejak A, Ruehland C. 2006. Symbioses between bacteria and gutless marine oligochaetes, p. 251–275. *In* Overmann, J (ed.), Progress in molecular and subcellular biology. Springer-Verlag Berlin, Berlin.

10. Eichinger I, Klepal W, Schmid M, Bright M. 2011. Organization and microanatomy of the *Sclerolinum contortum* trophosome (Polychaeta, Siboglinidae). Biol Bull 220:140–153.

11. Pasteris JD, Freeman JJ, Goffredi SK, Buck KR. 2001. Raman spectroscopic and laser scanning confocal microscopic analysis of sulfur in living sulfur-precipitating marine bacteria. Chem Geol 180:3–18.

12. White SN. 2009. Laser Raman spectroscopy as a technique for identification of seafloor hydrothermal and cold seep minerals. Chem Geol 259:240–252.

**Figure S1. Phylogenetic analysis of the delta- (A) and alphaproteobacterial (B) symbionts and associated bacteria of *Inanidrilus exumae* based on 16S rRNA gene sequences.** Sequences obtained in this study are framed with a red box (1444-1522 bp long), sequences from gutless phallodriline symbionts are highlighted in yellow. The consensus trees shown are based on maximum likelihood analysis. Branching orders that were not supported are shown as multifurcations. Scale bars represent 10% estimated phylogenetic divergence for non-multifurcation branches. assoc. bacterium refers to associated bacterium.

**Figure S2. Raman spectrogram of deparaffinized *Inanidrilus exumae* tissue and symbionts, and of *Ca.* Thiosymbion cells**. Raman spectrograms were baseline corrected and normalized. The 475 $cm^{-1}$ sulfur peak is indicated by a red arrow. The red circle in the phase contrast images shows were the sample was measured. All samples were analyzed with a D1 laser intensity filter and a 250 µm pinhole for 25 sec (15 sec in S2.2 and S2.4).

(**S2.1**) *I. exumae* section on an untreated glass slide. An additional possible sulfur peak is indicated by a blue arrow. (**S2.2**) *I. exumae* section on a $CaF_2$ slide. The background peak of $CaF_2$ (320 $cm^{-1}$) is indicated by a blue arrow. (**S2.3**) *I. exumae* host tissue, on an untreated glass slide, did not show peaks characteristic for sulfur. (A: muscle tissue, B: muscle tissue, C: core region). (**S2.4**) Fresh *Ca.* Thiosymbion cells on an untreated glass slide show three peaks indicative of S8 sulfur. **(S2.5)** Fixed *Ca.* Thiosymbion cells on a $CaF_2$ slide show only one sulfur peak at about 480 $cm^{-1}$. The $CaF_2$ background peak is visible at about 320 $cm^{-1}$.

Figure S1

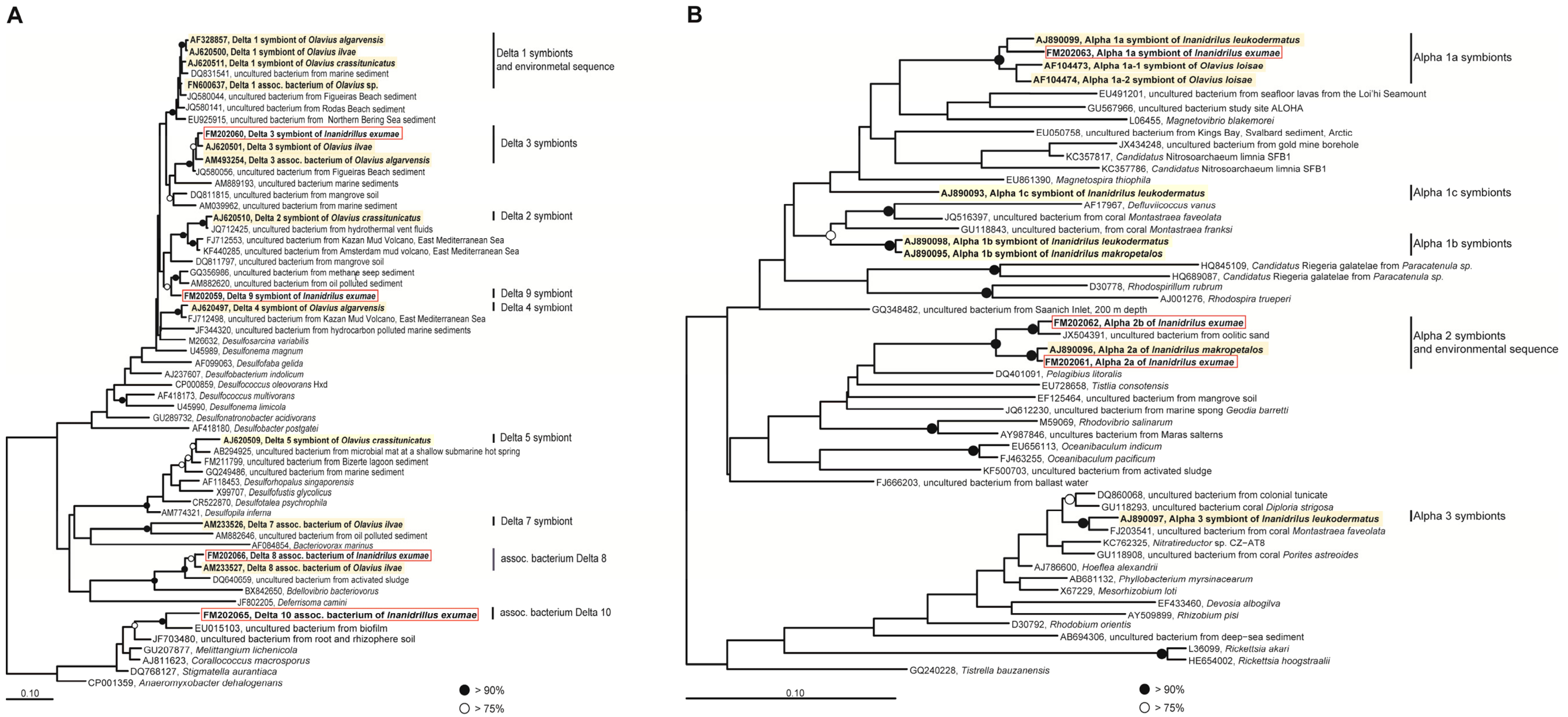
A
AF328857, Delta 1 symbiont of Olavius algarvensis
AJ620500, Delta 1 symbiont of Olavius ilvae
AJ620511, Delta 1 symbiont of Olavius crassitunicatus
DQ831541, uncultured bacterium from marine sediment
FN600637, Delta 1 assoc. bacterium of Olavius sp.
JQ580044, uncultured bacterium from Figueiras Beach sediment
JQ580141, uncultured bacterium from Rodas Beach sediment
EU925915, uncultured bacterium from Northern Bering Sea sediment
FM202060, Delta 3 symbiont of Inanidrillus exumae
AJ620501, Delta 3 symbiont of Olavius ilvae
AM493254, Delta 3 assoc. bacterium of Olavius algarvensis
JQ580056, uncultured bacterium from Figueiras Beach sediment
AM889193, uncultured bacterium marine sediments
DQ811815, uncultured bacterium from mangrove soil
AM039962, uncultured bacterium from marine sediment
AJ620510, Delta 2 symbiont of Olavius crassitunicatus
JQ712425, uncultured bacterium from hydrothermal vent fluids
FJ712553, uncultured bacterium from Kazan Mud Volcano, East Mediterranean Sea
KF440285, uncultured bacterium from Amsterdam mud volcano, East Mediterranean Sea
DQ811797, uncultured bacterium from mangrove soil
GQ356986, uncultured bacterium from methane seep sediment
AM882620, uncultured bacterium from oil polluted sediment
FM202059, Delta 9 symbiont of Inanidrilus exumae
AJ620497, Delta 4 symbiont of Olavius algarvensis
FJ712498, uncultured bacterium from Kazan Mud Volcano, East Mediterranean Sea
JF344320, uncultured bacterium from hydrocarbon polluted marine sediments
M26632, Desulfosarcina variabilis
U45989, Desulfonema magnum
AF099063, Desulfofaba gelida
AJ237607, Desulfobacterium indolicum
CP000859, Desulfococcus oleovorans Hxd
AF418173, Desulfococcus multivorans
U45990, Desulfonema limicola
GU289732, Desulfonatronobacter acidivorans
AF418180, Desulfobacter postgatei
AJ620509, Delta 5 symbiont of Olavius crassitunicatus
AB294925, uncultured bacterium from microbial mat at a shallow submarine hot spring
FM211799, uncultured bacterium from Bizerte lagoon sediment
GQ249486, uncultured bacterium from marine sediment
AF118453, Desulforhopalus singaporensis
X99707, Desulfofustis glycolicus
CR522870, Desulfotalea psychrophila
AM774321, Desulfopila inferna
AM233526, Delta 7 assoc. bacterium of Olavius ilvae
AM882646, uncultured bacterium from oil polluted sediment
AF084854, Bacteriovorax marinus
FM202066, Delta 8 assoc. bacterium of Inanidrilus exumae
AM233527, Delta 8 assoc. bacterium of Olavius ilvae
DQ640659, uncultured bacterium from activated sludge
BX842650, Bdellovibrio bacteriovorus
JF802205, Deferrisoma camini
FM202065, Delta 10 assoc. bacterium of Inanidrillus exumae
EU015103, uncultured bacterium from biofilm
JF703480, uncultured bacterium from root and rhizophere soil
GU207877, Melittangium lichenicola
AJ811623, Corallococcus macrosporus
DQ768127, Stigmatella aurantiaca
CP001359, Anaeromyxobacter dehalogenans
Delta 1 symbionts
and environmetal sequence
Delta 3 symbionts
Delta 2 symbiont
Delta 9 symbiont
Delta 4 symbiont
Delta 5 symbiont
Delta 7 symbiont
assoc. bacterium Delta 8
assoc. bacterium Delta 10
0.10
> 90%
> 75%
B
AJ890099, Alpha 1a symbiont of Inanidrilus leukodermatus
FM202063, Alpha 1a symbiont of Inanidrilus exumae
AF104473, Alpha 1a-1 symbiont of Olavius loisae
AF104474, Alpha 1a-2 symbiont of Olavius loisae
EU491201, uncultured bacterium from seafloor lavas from the Loi'hi Seamount
GU567966, uncultured bacterium study site ALOHA
L06455, Magnetovibrio blakemorei
EU050758, uncultured bacterium from Kings Bay, Svalbard sediment, Arctic
JX434248, uncultured bacterium from gold mine borehole
KC357817, Candidatus Nitrosoarchaeum limnia SFB1
KC357786, Candidatus Nitrosoarchaeum limnia SFB1
EU861390, Magnetospira thiophila
AJ890093, Alpha 1c symbiont of Inanidrilus leukodermatus
AF17967, Defluviicoccus vanus
JQ516397, uncultured bacterium from coral Montastraea faveolata
GU118843, uncultured bacterium, from coral Montastraea franksi
AJ890098, Alpha 1b symbiont of Inanidrilus leukodermatus
AJ890095, Alpha 1b symbiont of Inanidrilus makropetalos
HQ845109, Candidatus Riegeria galatelae from Paracatenula sp.
HQ689087, Candidatus Riegeria galatelae from Paracatenula sp.
D30778, Rhodospirillum rubrum
AJ001276, Rhodospira trueperi
GQ348482, uncultured bacterium from Saanich Inlet, 200 m depth
FM202062, Alpha 2b of Inanidrilus exumae
JX504391, uncultured bacterium from oolitic sand
AJ890096, Alpha 2a of Inanidrilus makropetalos
FM202061, Alpha 2a of Inanidrilus exumae
DQ401091, Pelagibius litoralis
EU728658, Tistlia consotensis
EF125464, uncultured bacterium from mangrove soil
JQ612230, uncultured bacterium from marine spong Geodia barretti
M59069, Rhodovibrio salinarum
AY987846, uncultures bacterium from Maras salterns
EU656113, Oceanibaculum indicum
FJ463255, Oceanibaculum pacificum
KF500703, uncultured bacterium from activated sludge
FJ666203, uncultured bacterium from ballast water
DQ860068, uncultured bacterium from colonial tunicate
GU118293, uncultured bacterium coral Diploria strigosa
AJ890097, Alpha 3 symbiont of Inanidrilus leukodermatus
FJ203541, uncultured bacterium from coral Montastraea faveolata
KC762325, Nitratireductor sp. CZ−AT8
GU118908, uncultured bacterium from coral Porites astreoides
AJ786600, Hoeflea alexandrii
AB681132, Phyllobacterium myrsinacearum
X67229, Mesorhizobium loti
EF433460, Devosia albogilva
AY509899, Rhizobium pisi
D30792, Rhodobium orientis
AB694306, uncultured bacterium from deep−sea sediment
L36099, Rickettsia akari
HE654002, Rickettsia hoogstraalii
GQ240228, Tistrella bauzanensis
Alpha 1a symbionts
Alpha 1c symbionts
Alpha 1b symbionts
Alpha 2 symbionts
and environmental sequence
Alpha 3 symbionts
0.10
> 90%
> 75%

Figure S2.1

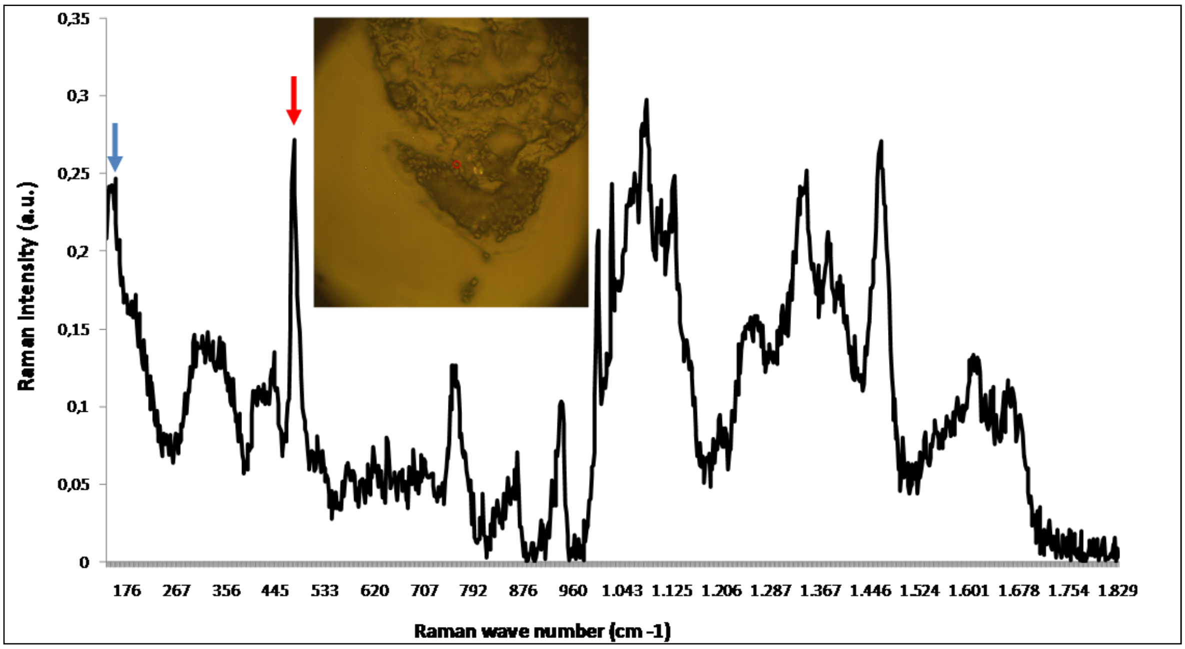


Figure S2.2

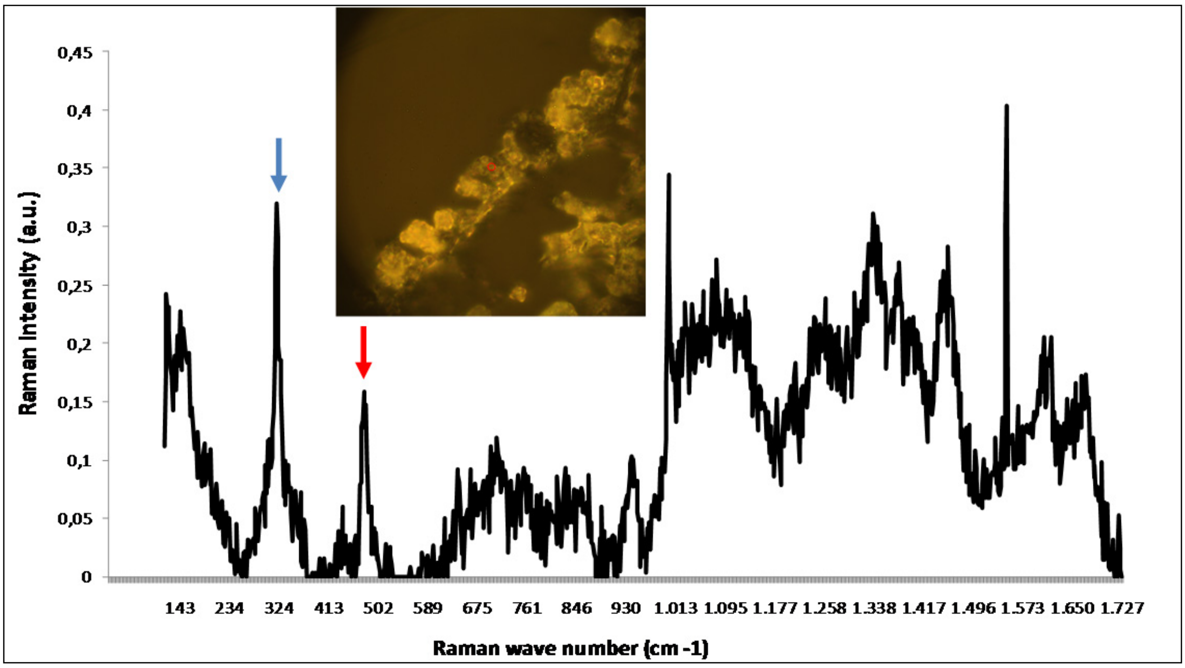

Figure S2.3

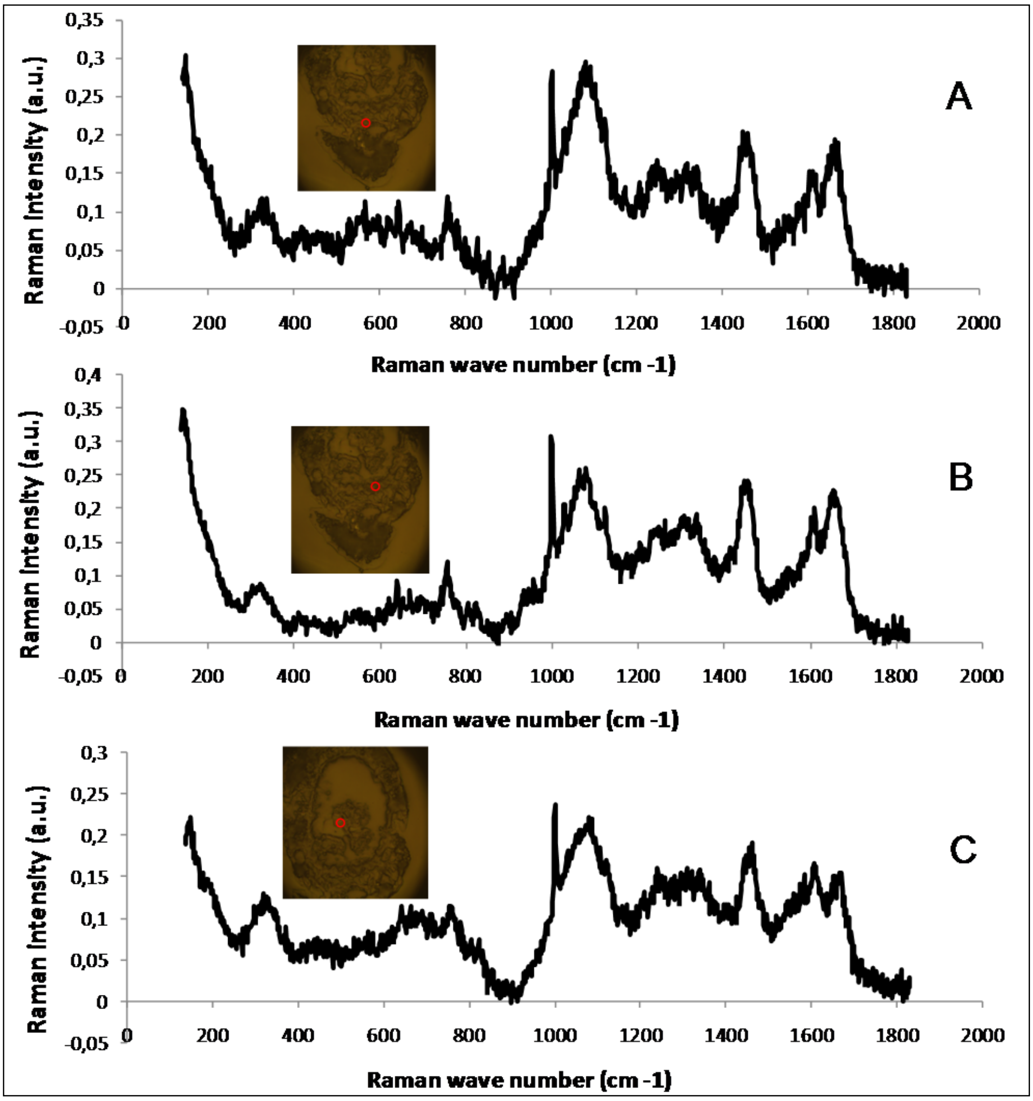

A
B
C
Raman Intensity (a.u.)
Raman wave number (cm -1)
0,35
0,3
0,25
0,2
0,15
0,1
0,05
0
-0,05
0,4
0
200
400
600
800
1000
1200
1400
1600
1800
2000

Figure S2.4

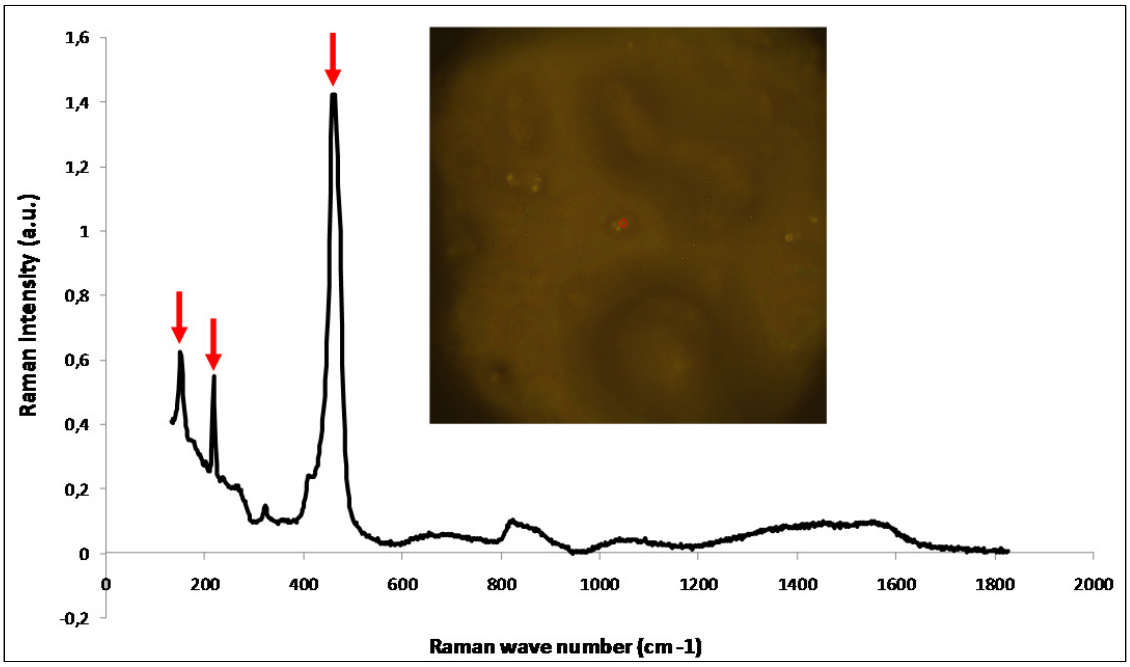


Figure S2.5

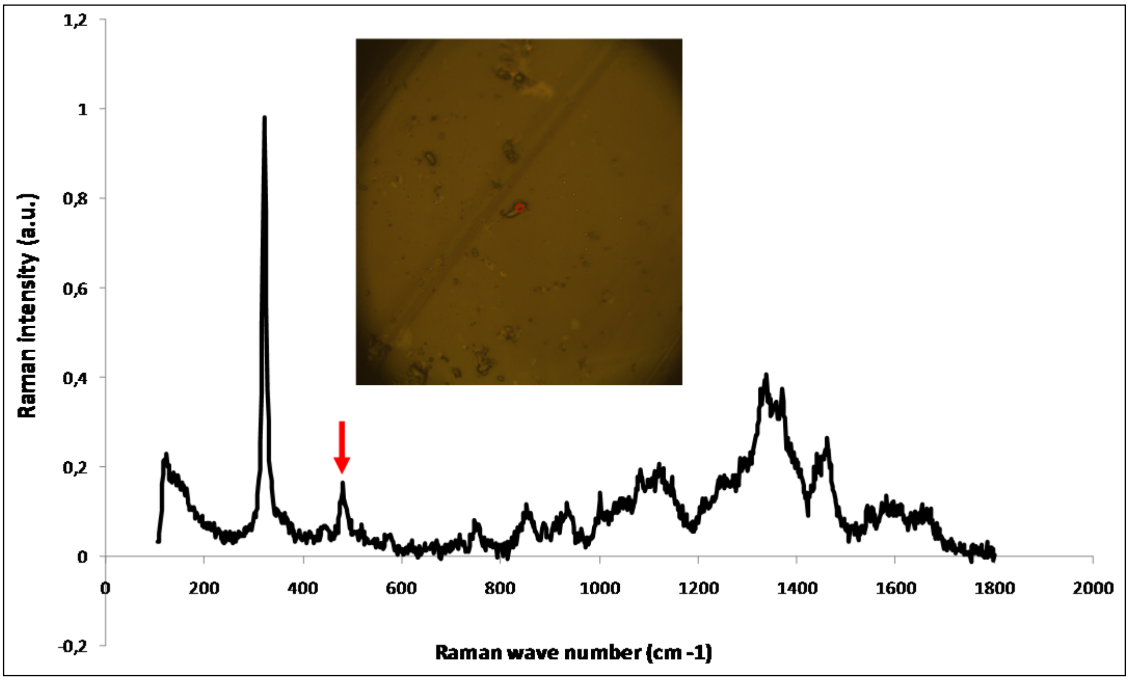

Supplement: Supplemental material [file AEM.02267-17_zam007188392s1.pdf]
